# Preparation of a Ni_3_Sn_2_ alloy-type anode embedded in carbon nanofibers by electrospinning for lithium-ion batteries

**DOI:** 10.1039/d2ra05734d

**Published:** 2022-09-29

**Authors:** Nurbol Ibadulla, Ayaulym Belgibayeva, Arailym Nurpeissova, Zhumabay Bakenov, Gulnur Kalimuldina

**Affiliations:** National Laboratory Astana Kabanbay Batyr Ave. 53 Nur-Sultan 010000 Kazakhstan zbakenov@nu.edu.kz; Department of Chemical and Materials Engineering, School of Engineering and Digital Sciences, Nazarbayev University Kabanbay Batyr Ave. 53 Nur-Sultan 010000 Kazakhstan; Department of Mechanical and Aerospace Engineering, School of Engineering and Digital Sciences, Nazarbayev University Kabanbay Batyr Ave. 53 Nur-Sultan 010000 Kazakhstan gkalimuldina@nu.edu.kz

## Abstract

A pure-phase Ni_3_Sn_2_ intermetallic alloy encapsulated in a carbon nanofiber matrix (Ni_3_Sn_2_@CNF) was successfully prepared by electrospinning and applied as anode for lithium-ion batteries. The physical and electrochemical properties of the Ni_3_Sn_2_@CNF were compared to that of pure CNF. The resultant Ni_3_Sn_2_@CNF anode produced a high initial discharge capacity of ∼1300 mA h g^−1^, later stabilizing and retaining ∼350 mA h g^−1^ (*vs.* 133 mA h g^−1^ for CNF) after 100 cycles at 0.1C. Furthermore, even at a high current density of 1C, it delivered a high initial discharge capacity of ∼1000 mA h g^−1^, retaining ∼313 mA h g^−1^ (*vs.* 66 mA h g^−1^ for CNF) at the 200th cycle. The superior electrochemical properties of the Ni_3_Sn_2_@CNF over CNF were attributed to the presence of electrochemically active Sn and decreased charge-transfer resistance with the alloy encapsulation, as confirmed from cyclic voltammetry (CV) and electrochemical impedance spectroscopy (EIS) results. Finally, post-mortem field-emission scanning electron microscopy (FE-SEM) images proved the preservation of the carbon nanofibers and the alloy after cycling, confirming the successful accommodation of the volume changes during the alloying/dealloying reactions of Sn in the Ni_3_Sn_2_@CNF.

## Abstract 2

Ni_3_Sn_2_ intermetallic alloy encapsulated in a carbon nanofiber matrix (Ni_3_Sn_2_@CNF) was obtained by electrospinning. The Ni_3_Sn_2_@CNF anode produced a high initial discharge capacity of ∼1300 mA h g^−1^, later retaining ∼350 mA h g^−1^ after 100 cycles.

## Graphical abstract

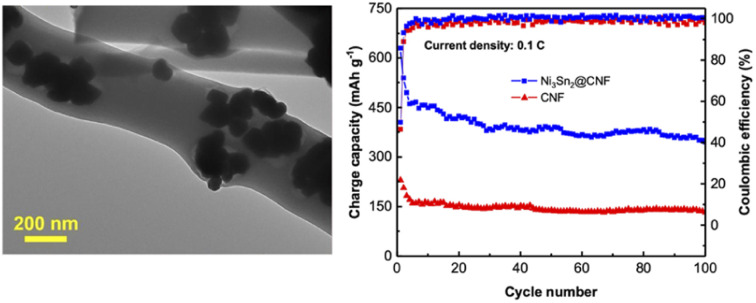

## 1. Introduction

With the demand for the advancement of portable technology and electric cars, the need for devices and machines with advanced characteristics has risen. These characteristics include but are not limited to safety, longevity, stability and ease of operation. Among these and many other properties, the cycle life and stability of the device are by far of the utmost importance for most people. The battery of the device directly affects the above mentioned characteristics. While there are many types of rechargeable batteries, Li-ion technology has found a broad use in portable electronics and electric vehicles since being first commercialized by Sony in 1991.^1–3^ The present Li-ion batteries (LIBs) mostly consist of lithiated transition metal oxides as a cathode material, and graphite and Li_4_Ti_5_O_12_ as an anode material. The mentioned anode materials deliver a theoretical capacity of ∼372 mA h g^−1^ and ∼175 mA h g^−1^.^4,5^ These capacities do not satisfy the current trends in the consumer electronics industry, which is more and more focused on battery capacity.

Alloying type tin (Sn) is attracting more attention as an alternative anode material for LIBs, owing to its high theoretical capacity (∼994 mA h g^−1^).^6^ However, its wide application is challenging because of large irreversible capacity, huge capacity fading and poor cycling, originating from a huge volume expansion (up to ∼300%) and electrode pulverization when Li^+^ ions are inserted and de-inserted.^7,8^ One approach to suppressing the volume expansion is coupling Sn with electrochemically inactive components, forming intermetallic alloy materials.^9^ During charging and discharging at the applied potential range, Sn interacts with Li^+^, while the inactive component keeps the structural stability and high electrical conductivity, thus enhancing the performance of the anode.^10,11^ Among different metals, Ni is widely studied as an inactive additive for Sn anode owing to its abundance in nature and low cost compared to Co and noble metals.^12^ Sn/Ni intermetallic alloys of different compositions (Ni_*x*_Sn_*y*_, *e.g.* Ni_3_Sn_4_ and Ni_3_Sn_2_, with theoretical capacities of ∼725 mA h g^−1^ and ∼570 mA h g^−1^, respectively) demonstrated lower volume expansion and better capacity retention compared to Sn alone.^13–17^

Enclosing the Sn with carbon is another common way to overcome its volume expansion. This has been implemented in a variety of ways such as encapsulation in hollow-shell, yolk–shell, core–shell spheres, core–shell nanotubes/nanofibers, suspension in a carbon matrix, *etc.*^18–21^ Along with the suppression of the volume expansion of Sn, coupling it with carbon overcomes the limited capacity problem of the carbon-based anodes. On the other hand, the combined effect of the Sn-based intermetallic alloy formation and carbon-encapsulation on the electrochemical properties of the composite anode has been barely studied.

Among different synthesis methods, electrospinning is a straightforward and cost-effective technique, which, in combination with heat treatments, allows the simultaneous *in situ* synthesis of various inorganic compounds and their encapsulation within the carbon nanofiber matrix.^22^ Furthermore, the prepared fibrous structure allows further improvement of the electrochemical properties of the electrode by shortening the Li^+^ diffusion pathway during the charge–discharge processes.^23^ For instance, Zhan *et al.* prepared free-standing and binder-free anode (FeP_2_@carbon nanofibers) using the electrospinning method.^24^ Authors could confine the well-dispersed FeP_2_ nanoparticles and amorphous phosphorus in the carbon nanofiber skeleton. As a result, they could improve the anode's kinetics and reduce the volume expansion during lithiation/delithiation. On the other hand, there are only a few works synthesizing Sn-based carbon nanofibers by electrospinning.^25–27^ In Yang *et al.* 's work, electrospinning is described as a practical approach for forming *in situ* composition between Sn and C. This can effectively suppress the volume change issues in the alloying reaction and decrease Sn nanoparticles agglomeration.^25^

In this work, we have prepared a pure-phase Ni_3_Sn_2_ intermetallic alloy encapsulated in CNF (Ni_3_Sn_2_@CNF) by electrospinning technique, using tin(ii) chloride dihydrate (SnCl_2_·2H_2_O) and nickel(ii) oxide (NiO) as Sn and Ni source, respectively. The electrochemical properties of the Ni_3_Sn_2_@CNF have been characterized as anode material for LIBs. Furthermore, the physical and electrochemical properties of the Ni_3_Sn_2_@CNF have been compared to that of pure CNF.

## 2. Experimental

### 2.1 Chemicals

Polyacrylonitrile (PAN) powder (average MW = 150 000 g mol^−1^) from J & K Scientific Ltd, *N*,*N*-dimethylformamide (DMF), SnCl_2_·2H_2_O, and NiO (nanopowder, <50 nm) from Aldrich Chemical Inc were used. All the chemicals were used as-received without further purification.

### 2.2 Preparation

The electrospinning solution for the synthesis of the Ni_3_Sn_2_@CNF was prepared by dissolving 1.2313 g of PAN in 15 mL of DMF and mixing it with 0.45 g of NiO and 0.8325 g of SnCl_2_·2H_2_O. The mixture was stirred at ambient temperature for more than 15 h. The mixture then was used for the electrospinning which was performed at a high voltage of 15 kV and a flow rate of 0.5 mL h^−1^. The electrospun fibers were collected on a static flat collector at a distance of 16 cm from the nozzle and dried in a vacuum oven at 60 °C overnight. Finally, the dried fibers were stabilized at 265 °C for 2 h in the compressed air atmosphere and then carbonized at 700 °C for 2.5 h in the Ar atmosphere using a tubular furnace with a heating rate of 3 °C min^−1^.

As a reference and for comparison, 1.75 g of PAN dissolved in 15 mL of DMF (11 wt%) was also electrospun and subsequently stabilized and carbonized at the same experimental conditions to prepare a pure CNF.

### 2.3 Characterization

The crystal structure was analyzed by X-ray diffraction (XRD, SmartLab, Rigaku) analysis, using Cu Kα radiation (*λ* = 1.54,059 Å) at 40 kV and 30 mA over a 2*θ* range from 20 to 90° at a scan rate of 4 ° min^−1^. X-ray photoelectron spectroscopy (XPS, NEXSA, Thermo Scientific) with a monochromatic Al Kα source was also employed to confirm the molecular structure. The carbon 1s spectrum at ∼284.99 eV was used as a reference for calibration. Raman spectroscopy (LabRAM, Horiba) was performed to check the carbon structure. Field-emission scanning electron microscopy (FE-SEM, Crossbeam 540, Carl Zeiss) coupled with energy-dispersive X-ray spectroscopy (EDS) were employed to investigate morphology and distribution of the sample composition, while transmission electron microscopy (TEM, JEM-1400 Plus, JEOL) was utilized to confirm its microstructure. CHNS analysis (CHNS-O, UNICUBE, Elementar) was used to determine the carbon content.

### 2.4 Electrochemical investigation

The electrochemical properties of the synthesized samples were characterized in CR2032 coin-type half-cells assembled in an argon-filled glove box (MBraun Inc). The working electrodes were coated onto Cu foil and composed of the active material (Ni_3_Sn_2_@CNF or CNF), binder (polyvinylidene difluoride, PVDF) and conductive agent (acetylene black, AB) in 90 : 5 : 5 weight ratio, respectively. The areal mass loading of electrodes was about 1.5–2 mg cm^−2^. Metallic lithium was used as both counter and reference electrodes. A Celgard 2400 microporous polypropylene membrane was used as a separator. The electrolyte was composed of 1 M LiPF_6_ in a mixture of ethylene carbonate/ethyl-methyl carbonate/dimethyl carbonate (EC/EMC/DMC, 1 : 1 : 1 vol%). The assembled cells were tested galvanostatically on a multi-channel battery testing system (Neware Battery tester, Neware Co.) at different current densities (1C = 570 mA g^−1^ for Ni_3_Sn_2_@CNF, and 372 mA g^−1^ for CNF), between the cut-off potentials of 0.01 and 1.5 V *vs.* Li/Li^+^. The capacities were calculated based on the mass of the composite. Cyclic voltammetry (CV) was performed using a VMP3 potentiostat/galvanostat (Bio-Logic Science Instrument Co.) at a scan rate of 0.1 mV s^−1^. Finally, electrochemical impedance spectroscopy (EIS) was performed after the 1st and 10th cycles at a frequency range from 10 mHz to 20 kHz with the altering voltage signal of 10 mV.

The morphology of the cycled Ni_3_Sn_2_@CNF was checked by post-mortem FE-SEM with EDS after 70 cycles at 0.1C.

## 3. Results and discussion

**Fig. 1:**
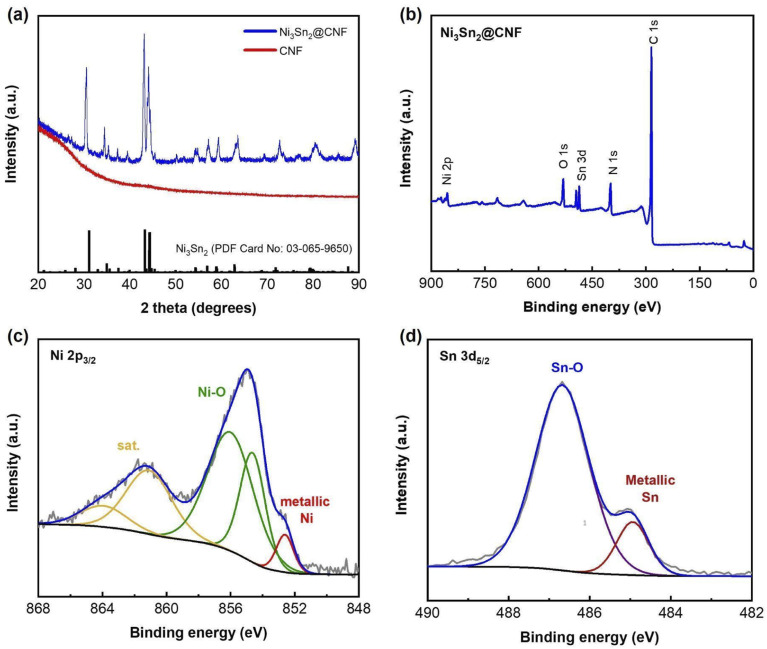
XRD patterns of both Ni_3_Sn_2_@CNF and CNF samples (a), XPS survey spectrum (b), Ni 2p_3/2_ (c) and Sn3d_5/2_ (d) XPS spectra of the Ni_3_Sn_2_@CNF.

The formation of the alloy after the carbonization of stabilized electrospun samples has been checked by XRD analysis. Fig. 1(a) shows the XRD patterns of the prepared samples, the pattern of pure CNF is given for comparison. Unlike the CNF, the pattern of the Ni_3_Sn_2_@CNF consists of sharp high-intensity peaks corresponding to Ni_3_Sn_2_ alloy (ICDD PDF card No 03-065-9650) without any impurities.

Fig. 1(b) shows the XPS survey spectrum of the Ni_3_Sn_2_@CNF composite. Apart from Ni, Sn, and C, the sample contains N, implying the formation of N-doped carbon beneficial for the improvement of electrical conductivity and electrochemical properties of the composite anode. The appearance of O may indicate surface oxidation during handling the sample in the ambient atmosphere, as no peaks of oxides have been observed on the XRD patterns (Fig. 1(a)). The Ni 2p_3/2_ spectrum in Fig. 1(c) has peaks, corresponding to metallic Ni state in intermetallic Ni_3_Sn_2_ at 852.61 eV, surface-oxidized Ni at 854.63 and 856.01 eV, and satellite at 861.1 eV.^27^ In the Sn3d_5/2_ spectrum (Fig. 1(d)), a major peak at 486.58 eV and a minor peak at 484.94 eV are observed, which are assigned to the surface-oxidized tin and intermetallic state of Ni_3_Sn_2_, respectively.^28^ These results confirm successful formation of the pure-phase Ni_3_Sn_2_ alloy in the prepared Ni_3_Sn_2_@CNF composite.

**Fig. 2:**
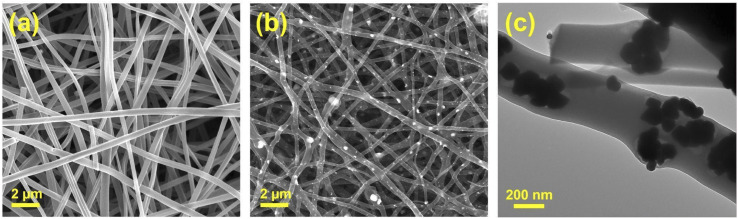
SEM image of the CNF (a), SEM image (b) and TEM image (c) of the Ni_3_Sn_2_@CNF.

Fig. 2(a) and (b) show the SEM images of the CNF and Ni_3_Sn_2_@CNF, respectively. It is clearly visible that the morphology of the Ni_3_Sn_2_@CNF (Fig. 2(b)) consists of nanoparticles uniformly scattered in, on and around the nanofibers, whereas the CNF (Fig. 2(a)) has smooth fibrous morphology without any particles. As confirmed from the TEM image of the Ni_3_Sn_2_@CNF in Fig. 2(c), aggregates of nanoparticles of almost similar sizes are well covered by an amorphous fibrous matrix, confirming the encapsulation of the Ni_3_Sn_2_ alloy particles inside the carbon nanofibers.

**Fig. 3:**
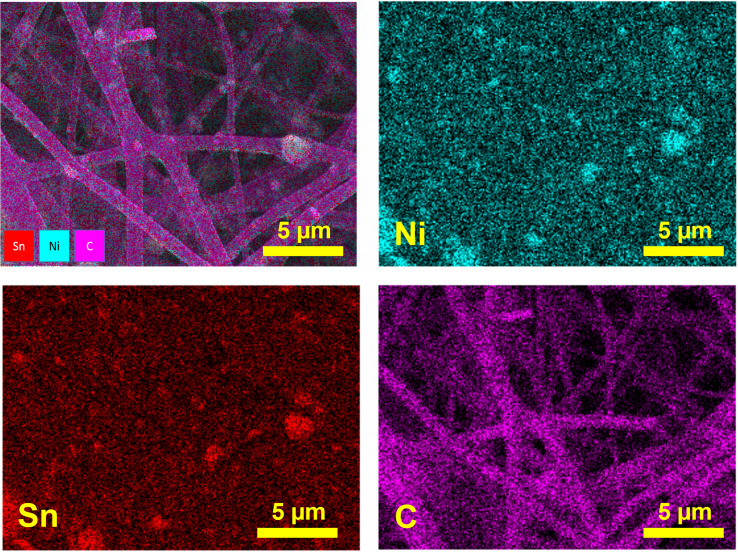
Elemental mapping of the Ni_3_Sn_2_@CNF.

Fig. 3 shows EDS elemental mapping of the Ni_3_Sn_2_@CNF. The mapping of Ni nanoparticles well overlaps with that of Sn nanoparticles and possess uniform distribution throughout the carbon nanofibers, reconfirming the encapsulation of Ni_3_Sn_2_ alloy in the carbon nanofiber matrix.

CHNS analysis has been carried out in order to determine the carbon content in the Ni_3_Sn_2_@CNF sample and it accounted for 58 wt% N-doped carbon.

**Fig. 4:**
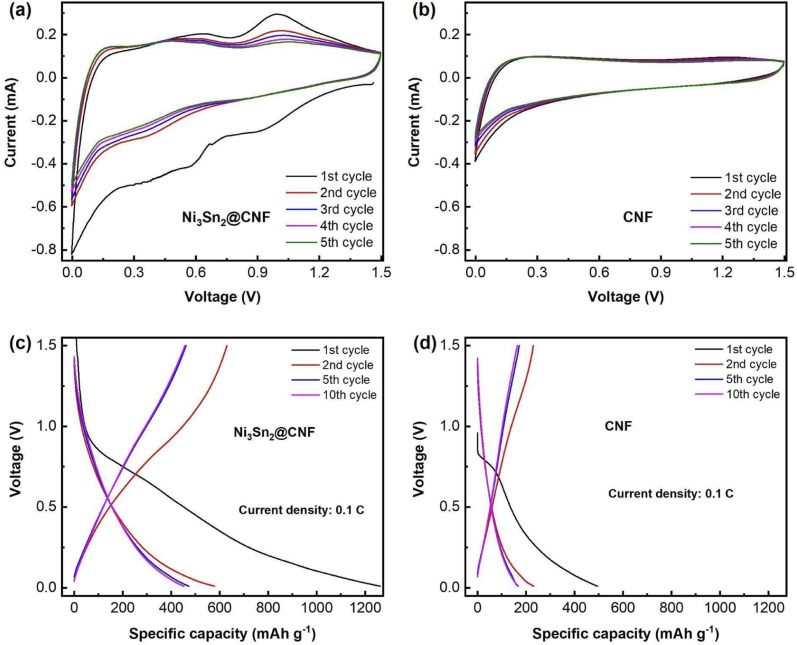
CV curves of the Ni_3_Sn_2_@CNF (a) and CNF (b) & charge–discharge profiles of the Ni_3_Sn_2_@CNF (c) and CNF (d) at 0.1C.

Fig. 4(a) and (b) show CV profiles of the Ni_3_Sn_2_@CNF and CNF, respectively. Unlike CNF, the curves of the Ni_3_Sn_2_@CNF contain several redox couples that correspond to the multi-step alloying/dealloying of Sn in Ni_3_Sn_2_ with Li^+^ upon cycling. The initial CV curve of the Ni_3_Sn_2_@CNF is different from consequent curves, probably because of the activation of the alloy accompanied by changes in the electrodes and formation of the solid electrolyte interphase (SEI) layer.^29^ The whole reaction is expected to proceed as follows:^30^

1 First charge: Ni_3_Sn_2_ + 8.8 Li^+^ + 8.8e^−^ → 2Li_4.4_Sn + 3Ni

2 Reversible charge: Li_4.4_Sn ↔ Sn + 4.4Li^+^ + 4.4e^−^

3 Reversible discharge: Sn + 4.4Li^+^ + 4.4e^−^ → Li_4.4_Sn

Reaction (1) occurs in the first charge accompanied by irreversible activation of Ni_3_Sn_2_@CNF, whereas reactions (2) and (3) operate in succession in the following cycles.

Fig. 4(c) and (d) show the potential profiles of the Ni_3_Sn_2_@CNF and CNF, respectively. As a result of the activation of the material, formation of the SEI layer, and alloying of Sn in the Ni_3_Sn_2_@CNF, the composite has a high initial discharge capacity of ∼1300 mA h g^−1^, which is 2.5 times higher than that of CNF. The initial charge curve of the Ni_3_Sn_2_@CNF in Fig. 4(c), contains two broad plateaus at ∼0.55 V and 0.8 V, corresponding to the reversible dealloying and extraction process of Li^+^ from the lithiated Sn in Ni_3_Sn_2_, contributing to the reversible capacity upon cycling. The potential profiles are in good agreement with the CV curves.

**Fig. 5:**
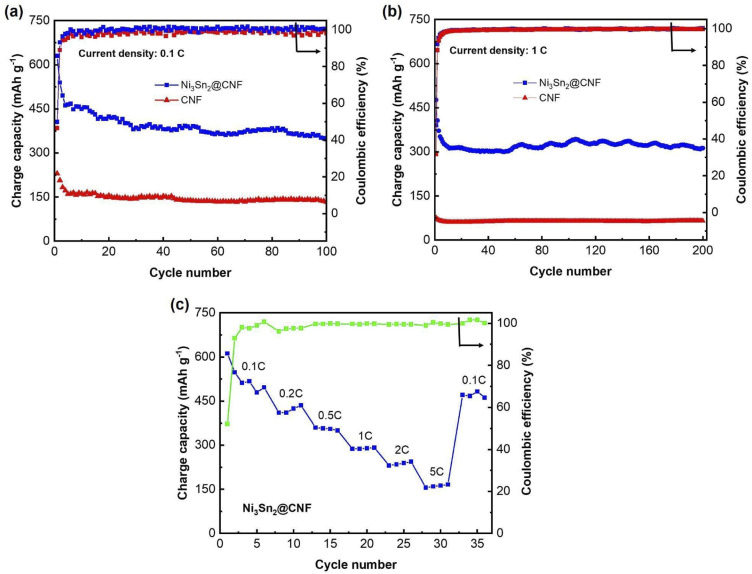
Cycle performance of the Ni_3_Sn_2_@CNF and CNF at 0.1C (a), at 1C (b) & rate capability test (c) of the Ni_3_Sn_2_@CNF.

Fig. 5(a) and (b) show the cycle performance of the Ni_3_Sn_2_@CNF and CNF at 0.1C and 1C, respectively. The Ni_3_Sn_2_@CNF has relatively good cyclability, retaining ∼350 mA h g^−1^ charge capacity after 100 cycles at a current density of 0.1C. In comparison, the CNF retains only ∼133 mA h g^−1^ at 100th cycle. The difference in charge capacities becomes even higher when the current density is increased to 1C. Thus, the Ni_3_Sn_2_@CNF retains 313 mA h g^−1^ charge capacity, while CNF retains only 66 mA h g^−1^ at 200th cycle at 1C.

Fig. 5(c) shows the rate capability of the Ni_3_Sn_2_@CNF electrode. Starting at ∼550 mA h g^−1^ at 0.1C, it showed a capacity of slightly over 400 mA h g^−1^, ∼370 mA h g^−1^, ∼300 mA h g^−1^, ∼230 mA h g^−1^, and ∼180 mA h g^−1^ at current densities of 0.2C, 0.5C, 1C, 2C and 5C, respectively. Finally, when the current density was brought back to 0.1C, a high capacity of ∼500 mA h g^−1^ could be recovered.

**Fig. 6:**
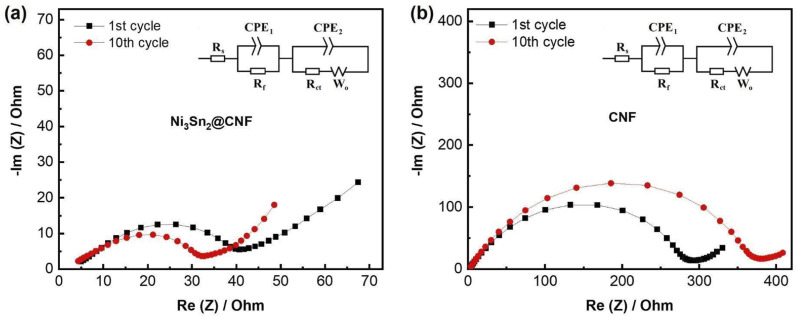
Nyquist plots of the Ni_3_Sn_2_@CNF (a) and CNF (b) measured after 1st and 10th cycles. Insets: equivalent circuit models.

EIS was performed to measure the cell resistance with prepared electrodes after the 1st and 10th cycles (Fig. 6). Nyquist plots of cells with both Ni_3_Sn_2_@CNF and CNF electrodes have the same features, namely one prolonged semicircle in the medium frequency that is usually attributed to the combined resistance from SEI layer (*R*_f_) and charge transfer (*R*_ct_), and an inclined line at low frequency region responsible for Li^+^ diffusion in the bulk of the electrode. The calculated resistance values are summarized in Table 1. The *R*_ct_ in Ni_3_Sn_2_@CNF cell is almost 10 and 16 times lower than that of CNF after the 1st and 10th cycles, respectively. The smaller *R*_ct_ in the Ni_3_Sn_2_@CNF cell is attributed to the higher electrical conductivity contributed by intermetallic alloy. Furthermore, the presence of the intermetallic alloy could catalyze the formation of a SEI layer with low resistance, which remains stable over cycles due to the unique structure of the Ni_3_Sn_2_@CNF.

**Table 1:** Fitting results of the Nyquist plots of the Ni_3_Sn_2_@CNF and CNF

	1st cycle			10th cycle		
	*R*_s_, Ω	*R*_f_, Ω	*R*_ct_, Ω	*R*_s_, Ω	*R*_f_, Ω	*R*_ct_, Ω
Ni_3_Sn_2_@CNF	4.82	6.25	27.60	3.92	6.03	20.46
CNF	4.14	25.45	241.8	4.17	27.91	320.9

**Fig. 7:**
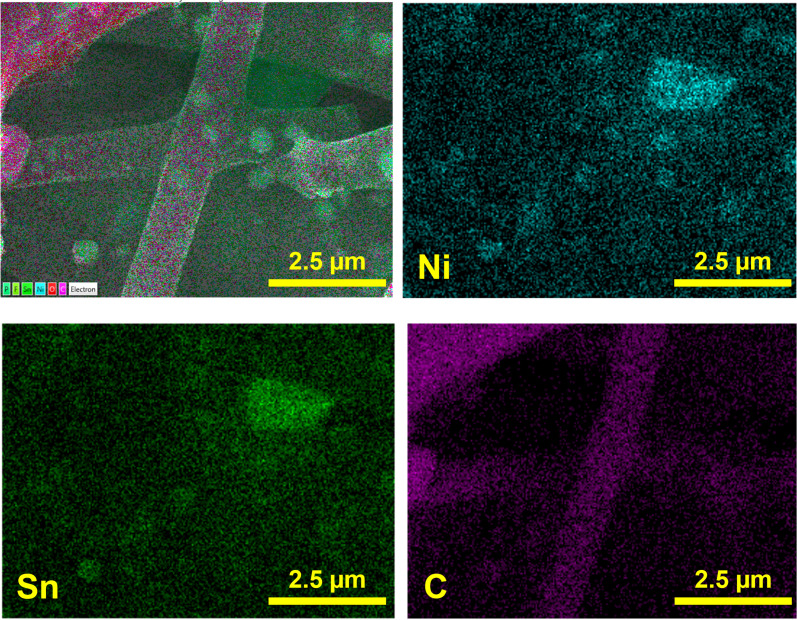
Elemental mapping of the Ni_3_Sn_2_@CNF after 70 cycles at 0.1C.

The morphology of the sample after cycling has been analyzed by post-mortem SEM and SEM-EDS after 70 cycles. Fig. 7 shows SEM image and elemental mapping of the Ni_3_Sn_2_@CNF after 70 cycles at 0.1C. The sample has preserved its fibrous morphology with encapsulation of alloy nanoparticles within the carbon fiber matrix, confirming the successful accommodation of the volume changes during the alloying/dealloying reactions of Sn in the Ni_3_Sn_2_@CNF and benefiting its electrochemical stability.

## 4. Conclusions

In conclusion, an anode in the form of an intermetallic alloy encapsulated in carbon nanofibers was obtained through the electrospinning method. The idea of synergistic effect of the Ni_3_Sn_2_ as an intermetallic buffering matrix that minimizes the volume expansion, and of encapsulation of this Ni_3_Sn_2_ in carbon nanofiber skeleton was successfully proven to be superior compared to the pure CNF. The alloy formation and the absence of impurities were confirmed by XRD and XPS techniques. The SEM, SEM-EDS and TEM images showed the encapsulation of the alloy in carbon nanofibers. The resultant composite had a high initial discharge capacity of ∼1300 mA h g^−1^, which is 2.5 times higher than that of CNF. Furthermore, at a high current density of 1C the Ni_3_Sn_2_@CNF retained 313 mA h g^−1^ charge capacity, while CNF retained only 66 mA h g^−1^ after 200 cycles. The superior electrochemical properties of the Ni_3_Sn_2_@CNF over CNF was attributed to the presence of electrochemically active Sn and decreased charge-transfer resistance with the alloy encapsulation, as confirmed from CV and EIS results. Finally, the post-mortem SEM and SEM-EDS images confirmed the preservation of the carbon nanofiber structure and the alloy, which was responsible for the stable cycle performance.

## Author contributions

N. I. synthesis and characterizations, writing; A. B., G. K., manuscript writing and editing, A. N., Z. B., G. K., supervision, editing.

## Conflicts of interest

The authors declare that they have no competing interests.

## Supplementary Material
